# *Aloe Vera* alleviates glyphosate-based herbicide hepatotoxicity in rats via anti-inflammatory and antioxidant actions

**DOI:** 10.3389/fvets.2025.1720985

**Published:** 2025-12-05

**Authors:** Nashwa Hamad, Ahmed A. Sharkawy, Nagwa Ibrahim, Amany Abdel Rahman Osman, Abdullah S. M. Aljohani, Heba F. Kamaly

**Affiliations:** 1 Department of Pathology, Faculty of Veterinary Medicine, Assiut University, Assiut, Egypt; 2 Department of Forensic Medicine and Toxicology, Faculty of Veterinary Medicine, Assiut University, Assiut, Egypt; 3 Department of Anatomy and Embryology, Faculty of Veterinary Medicine, Assiut University, Assiut, Egypt; 4 Chemistry Department, Faculty of Science, Assiut University, Assiut, Egypt; 5 Department of Medical Biosciences, College of Veterinary Medicine, Qassim University, Buraydah, Saudi Arabia

**Keywords:** *Aloe Vera*, glyphosate-based herbicide, hepatotoxicity, DNA damage, apoptosis

## Abstract

**Introduction:**

Glyphosate-Based Herbicide (GBHs) are widely used worldwide, this study investigated the ameliorative effect of *Aloe Vera* against GBHs hepatic toxicity in rats via anti-inflammatory and antioxidant Actions, with a focus on DNA damage and apoptosis.

**Methods:**

Twenty-four Sprague–Dawley rats were randomly classified into four groups: Group I, represented as a control. In group II, rats were administered 500 mg/kg of Roundup herbicide as one of GBHs. In Group III, rats were administered 200 mg/kg of *Aloe Vera*, and finally, in Group IV, rats were co-administered with 500 mg/kg of Roundup and 200 mg/kg of *Aloe Vera*, orally, three times a week for 6 weeks.

**Results and Discussion:**

Rats exposed to GBH showed significant oxidative stress, evidenced by elevated malondialdehyde (MDA) and decrease the reduced glutathione (GSH) levels, alongside increased the inflammatory markers (TNF-*α* and IL-1β). Liver function enzymes (ALT, AST, LDH) were markedly elevated, and comet assay results revealed substantial DNA fragmentation in hepatocytes. Histopathological findings and immunohistochemical overexpression of caspase-3 confirmed the liver pathological lesions and apoptosis, respectively. Co-administration of *Aloe Vera* with GBH significantly alleviated the biochemical, histopathological, and apoptotic alterations, but it did not fully reverse the DNA damage. These findings suggest that *Aloe Vera* may offer partial hepatoprotection against GBH toxicity through its antioxidant and anti-inflammatory properties.

## 1 Introduction

Glyphosate-based herbicides (GBHs) are the most used non-selective herbicide in agriculture, forestry, and weed control worldwide (1–3). Glyphosate residues were detected in the environment (4), food (5) and animal feed (6), thus their toxicity to livestock and the environment is a controversial topic (7).

Previously, glyphosate was considered safe for humans and animals (8). Contemporary studies indicates that exposure to glyphosate or its metabolites, even within established permissible limits, can induces oxidative stress through interference with mitochondrial oxidative phosphorylation and reactive oxygen species’ generation (9–12), which can alter the cellular lipids, proteins, and DNA, as well as activate the apoptosis pathways (13).

Glyphosate residues and metabolites act as environmental contaminants, and are frequently detected in food, feed, soil and water (14–16). Approximately 20% of ingested glyphosate is absorbed from the gastrointestinal tract (17), and 30–36% of the absorbed portion is distributed with high concentrations into bone, kidneys, and liver (18), which can exert different systemic effects (19). Finally, GBHs are excreted via feces and urine (20–22) and rarely accumulate in animals (15).

Aminomethyl phosphoric acid (AMPA) and formaldehyde are the main glyphosate metabolites. In animal tissues, enzymes can catalyze the formaldehyde to formic acid (23), which causes malfunction of metabolic acidosis and mitochondria (24).

Roundup® is the first choice formulation for weed control thanks to its potency, where it comprises mixtures of glyphosate and adjuvants to enhance the active ingredient uptake and translocation into plant tissues (25). Commercially, Roundup formulated with polyethoxylated tallow amine (POEA) surfactant with the isopropylamine salt (IPS) of glyphosate. POEA surfactant is responsible for the weed-killing ability of glyphosate, where it increases the permeability of glyphosate salt to the plant cells (26–28). Herbicide brands which contain previous surfactants may be more toxic and persistent than glyphosate alone (29, 30). Thus, Roundup has negative effects on various enzymes, which play essential roles in chemicals’ detoxification and metabolism in the liver (31–33). Saleh et al. (34) revealed the Roundup (glyphosate 48%) hebatotoxicity in albino rats through histopathological examination and biochemical analysis. Pandey et al. (35) observed the inflammatory effects of Herbicide Roundup (41% w/w glyphosate) in rat liver. Djaber et al. (36) and Djaber et al. (37) studied the oxidative stress effect and histopathological lesions of Roundup® TURBO, which contain glyphosate active ingredient in rat liver.

In 2015, due to various adverse effects of GBHs, the International Agency for Research on Cancer (IARC) classified them as a probable human carcinogen (Group 2A) (38). Hepatotoxicity, renal toxicity, neurotoxicity, reproductive toxicity, genotoxicity, endocrine disruption, and chronic diseases were reported on non-target organisms (12, 15, 20, 39–41). The liver and kidney were the main target organs related to Roundup consumption, distribution and bioaccumulation (42, 43). The previous studies were limited to the hepato-renal toxicity of low and ultralow doses of glyphosate in animals (41, 44, 45).

In this context, the liver is of special interest due to its vital role in xenobiotic metabolism (46, 47). Alleviation of the oxidative stress effects can be achieved through diet supplementation with effective antioxidants such as *Aloe Vera*. It belongs to the family Liliaceae and is considered one of the most popular medicinal plants which exhibit numerous pharmacological and therapeutic activities (48). *Aloe Vera* with wide geographical distribution acts as an anti-inflammatory, antioxidant, gout reducer, anti-cancer agent, antioxidant, antimicrobial, anti-diabetic, immunomodulatory, wound healer and gastroprotective agent (49–51), thanks to its antioxidants, vitamins, amino acids, enzymes, minerals, terpenoids, phenolics, glycosides of phenolics, polysaccharides, peptides, resins, alkaloids, essential oils, and chromones (52–54) bioactive phytoconstituent content.

The specific studies on *Aloe Vera*’s role against glyphosate toxicity are lacking, thus the current study acts as a first approach and aimed to detect the mitigating role of *Aloe Vera* on the hepatic toxicity induced by Roundup based herbicide.

## 2 Materials and methods

### 2.1 Ethical approval

The present experimental research had ethical approval with protocol No. 06/2025/0327 from the Faculty of Veterinary Medicine ethical committee, Assiut University, Egypt.

### 2.2 Chemicals

- Roundup® STAR 44.1% SL [N-(Phosphonomethyl) glycine, unspecified potassium salt], Monsanto Company, Belgium was purchased from the Central Agricultural Pesticide Laboratory, Giza, Egypt.
- *Aloe Vera* (200 mg/capsule) with ZIN: 518925 was bought from American NatureCity brand, Pharmavite Company, United States.

### 2.3 Experimental animals

The guidelines of laboratory animal care were applied during the experiment; 8 weeks old, 180–200 gram adult female Sprague–Dawley rats were procured from the Experimental Animal House at the Faculty of Veterinary Medicine, Assiut University. Rats were acclimatized to the surroundings with feed and water *ad libitum* for 2 weeks prior to the experiment under ideal conditions of housing in suitable polypropylene cages, temperature, lighting and humidity.

### 2.4 Experimental design

In the current experimental study, 24 adult female rats were divided into four groups (*n* = 6) as follows: Group I rats served as a control for the experiment. Group II rats were administered 500 mg/kg of Roundup (1/10 LD_50_) according to Pandey et al. (35). Group III rats were administered 200 mg/kg of *Aloe Vera*, and finally, group IV rats were administered 500 mg/kg of Roundup and 200 mg/kg of *Aloe Vera*. Roundup®-based herbicide and *Aloe Vera* in all treated groups were orally administered, by gavage, three times a week for 6 weeks after they were diluted in distilled water.

### 2.5 Samples collection and liver homogenate preparation

After 6 weeks, blood samples were collected in vacutainer tubes without anticoagulant from the medial canthus of the orbital cavity of rats. The sera were taken by centrifugation for 15 min. at 3500 rpm for liver enzymes evaluation.

Rats’ liver were dissected out immediately after their anesthetized with 100 mg/kg of sodium thiopental via IP injection according to Abd-Eldayem et al. (55). A piece of each liver (500 mg) was homogenized in 5 mL PBS, centrifuged, and the supernatants were obtained and stored at - 80 °C for estimation of oxidative stress and inflammatory markers. The rest of each liver was divided into 2 pieces. One piece was kept at -80 °C for a comet assay, whereas the other piece was immersed in neutral buffered formalin (10%) fixative for histopathological and immunohistochemical investigations.

### 2.6 Estimation of hepatic malondialdehyde and reduced glutathione

Determination of MDA and GSH levels in the liver homogenates’ supernatants was through using the standard assaying kits (Biodiagnostic, Giza, Egypt, Catalog Number: MD 25 28) and (Biodiagnostic, Giza, Egypt, Catalog Number: GR 25 10) respectively, according to the guidelines of the manufacturer.

### 2.7 Estimation of interleukin-1β and tumor necrosis factor-*α* in liver

Using an ELISA Kit, IL-1β (Elabscience, United States, Catalog No: E-EL-R0012) and TNF-α (Assay Pro, St. Charles, MO, United States, Catalog Number: ERT2010-1) were estimated in the hepatic tissue homogenate.

### 2.8 Estimation of liver function enzymes (ALT, AST, and LDH)

Liver enzyme (ALT and AST) levels in serum were assessed according to the instructions of the manufacturer of standard kits provided by Chema Diagnostica via Campania, Monsano, Italy. The enzyme-linked immunosorbent assay Kit provided by Abbexa Ltd., Cambridge, United Kingdom, was employed for assessing LDH serum levels following the included instructions.

### 2.9 Hepatocytes comet assay

The single-cell gel electrophoresis was conducted according to published methods (56, 57) with modifications in homogenization and lysing steps: 0.5 g of liver sample was minced, homogenized gently and centrifuged at 1500 rpm for 10 min. at 0 °C. Re-suspend the precipitate in chilled homogenized buffer and allow it to settle for 1-2 min. Lysing the embedded cells in lysing buffer at 4 °C for 120 min. Followed with unwinding, electrophoresis and microscopically examination of the slides with florescent microscope (Olympus BX-43, Japan) with green filter. Digital camera was used to capture the images and slides were read at a 300 fold of magnification.

### 2.10 Histopathological examination of liver tissues

Liver tissue specimens were gathered quickly after sacrificing the rats, fixed by using neutral buffered formalin (10%), processed, and then stained by Hematoxylin and eosin (H&E) stain for histopathological examination (58). Slides were inspected using a light microscope. Thereafter, a digital camera was employed to take photographs. A grading system between 0 and 4 was utilized on H&E-stained hepatic sections to assess the severity of histopathological lesions semiquantitatively (59).

### 2.11 Immunohistochemical evaluation of apoptotic cells using caspase-3

Caspase-3 was detected by immunohistochemistry using paraffin sections from the liver. The tissue sections were dewaxed, rehydrated, and then washed by distal water. Afterwards, antigen retrieval was done by boiling the slides for 20 min with citrate buffer (pH 6). The endogenous peroxidase actions were suppressed by 3% hydrogen peroxide. After that, sections were incubated in a humidified chamber with primary antibody for Caspase-3 (AB Clonal Technology Company) at 1:100 dilution for an entire night at 4 °C. Then the sections were incubated in Econo Tek HRP Conjugate for 30 min at room temperature and washed 4 times for 5 min each with phosphate-buffered saline. Thereafter, slides were incubated for 10 min in a mixture of DAB chromogen and DAB substrate to visualize the reaction. Sections were washed with tap water, counterstained with Meyer’s hematoxylin, dehydrated, cleared, and covered by DPX (60).

The intensity of caspase-3 immunolabeling was semi-quantitatively scored on 10 high-power fields/slide in 6 slides (number of sections/group). Grading system from 0 to 3 were followed, where 0: no staining, 1: weak staining, 2: moderate staining, and 3: strong staining. Furthermore, the area percentage of caspase-3 immune expression of was calculated utilizing ImageJ software (61, 62).

### 2.12 Statistical analysis

One-way analysis of variance (ANOVA) was utilized for statistical analysis of the results obtained followed by Tukey’s *post-hoc* test, employing GraphPad Prism 5 (GraphPad Software, CA, United States). For comparisons among the studied groups, data was expressed as the mean ± standard error of mean (SEM), and statistical significance was approved when the *p*-value < 0.05.

## 3 Results

### 3.1 Oxidative status

**Figure 1:**
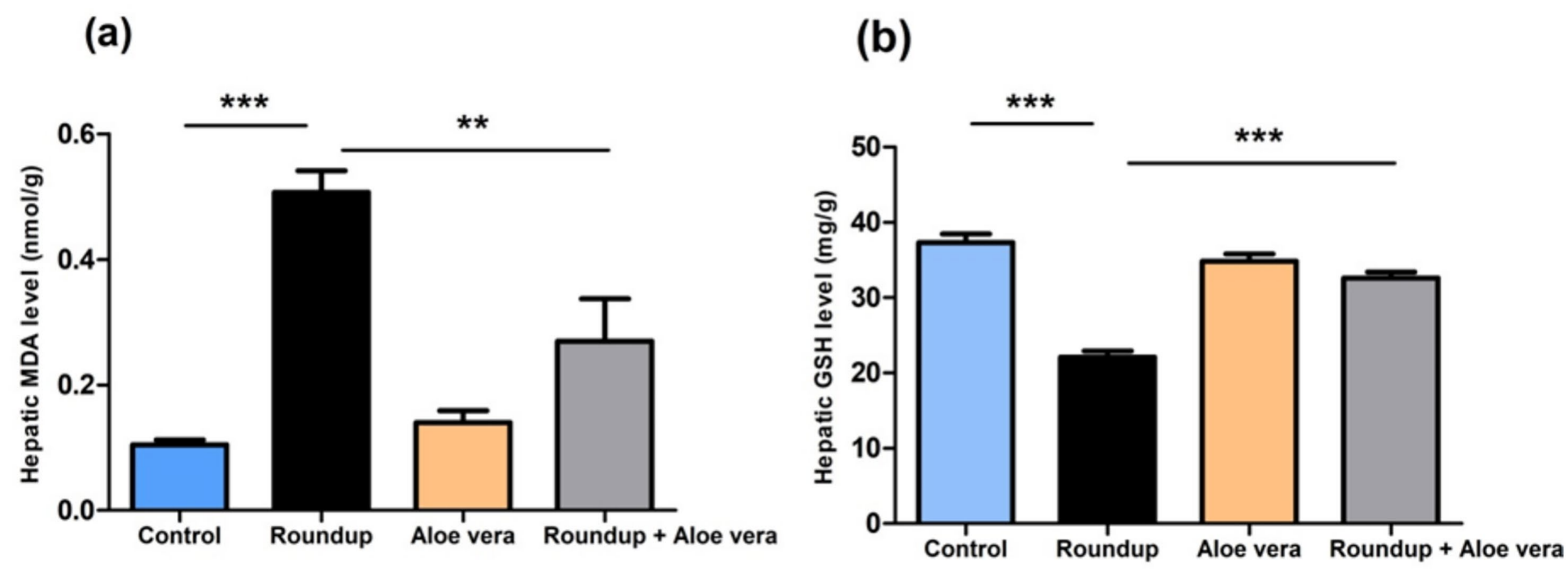
Effect of co-administration of Roundup and *Aloe Vera* on MDA and GSH of liver. **(a)** A significant decline in MDA and **(b)** a significant increment in reduced GSH in the Roundup + *Aloe Vera*-treated rats in comparison with the Roundup-treated rats. The results are expressed as mean ± SEM (*n* = 6/group). **p < 0.01 and ***p < 0.001.

The oxidative stress in the hepatic tissue of the Roundup-intoxicated rats was evidenced by the significant (*p* < 0.001) increase in tissue level of MDA and decrease in the level of GSH relative to the control. A non-significant difference was noticed in the *Aloe Vera* group versus the control. Rats co-administered *Aloe Vera* with Roundup displayed a significant decrease of hepatic MDA (*p* < 0.01) and an increase in GSH (*p* < 0.001) levels in comparison with the Roundup-exposed rats (Figure 1).

### 3.2 Hepatic IL-1β and TNF-*α*

**Figure 2:**
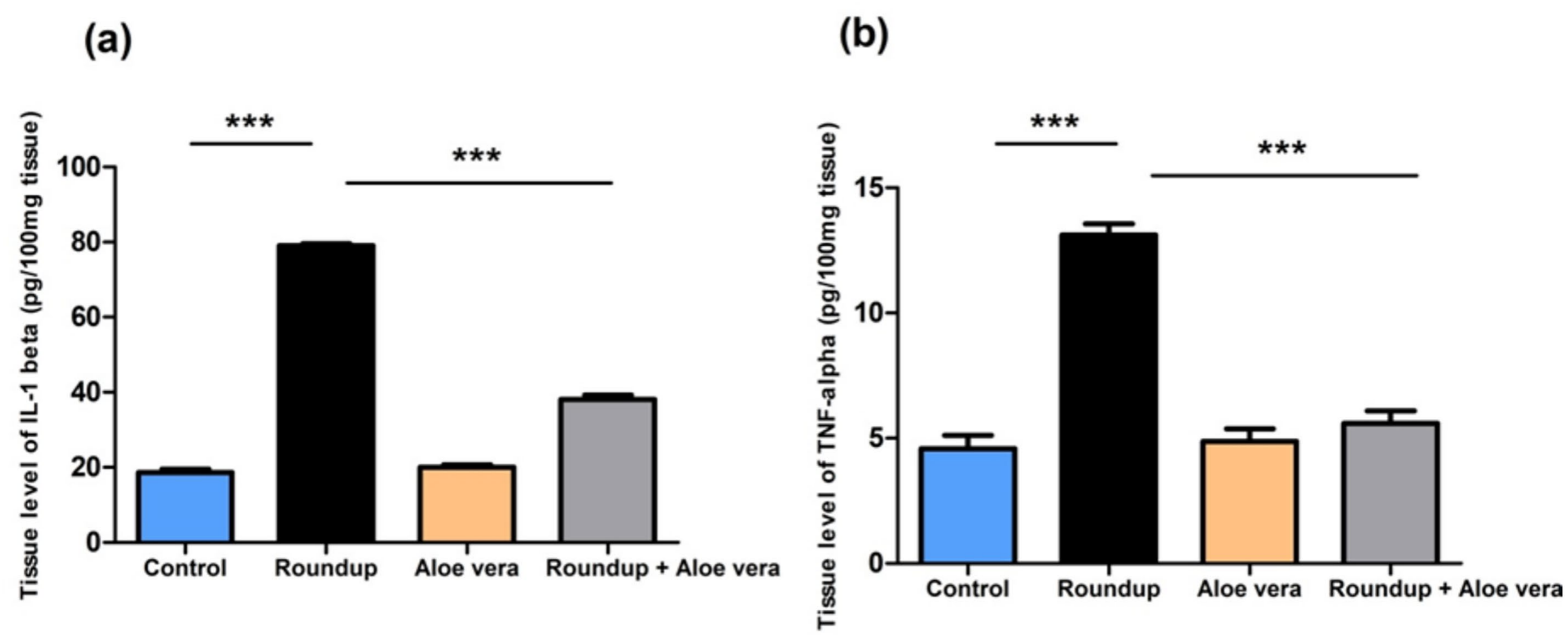
Effect of co-administration of Roundup and *Aloe Vera* on hepatic IL-1β and TNF-*α*. **(a)** IL-1β and **(b)** TNF-α levels showing a significant decrease in the Roundup + *Aloe Vera*-treated rats in comparison with the Roundup-exposed rats. ***p < 0.001.

The roundup-treated rats had a significant (*p* < 0.001) increase in the tissue IL-1β and TNF-α levels versus the control group. *Aloe Vera* showed non-significant results compared to the control. Co-administration of *Aloe Vera* with Roundup showed a significant (*p* < 0.001) diminishment in IL-1β and TNF-α levels in comparison with the Roundup-exposed rats (Figure 2).

### 3.3 Liver function enzymes

**Figure 3:**
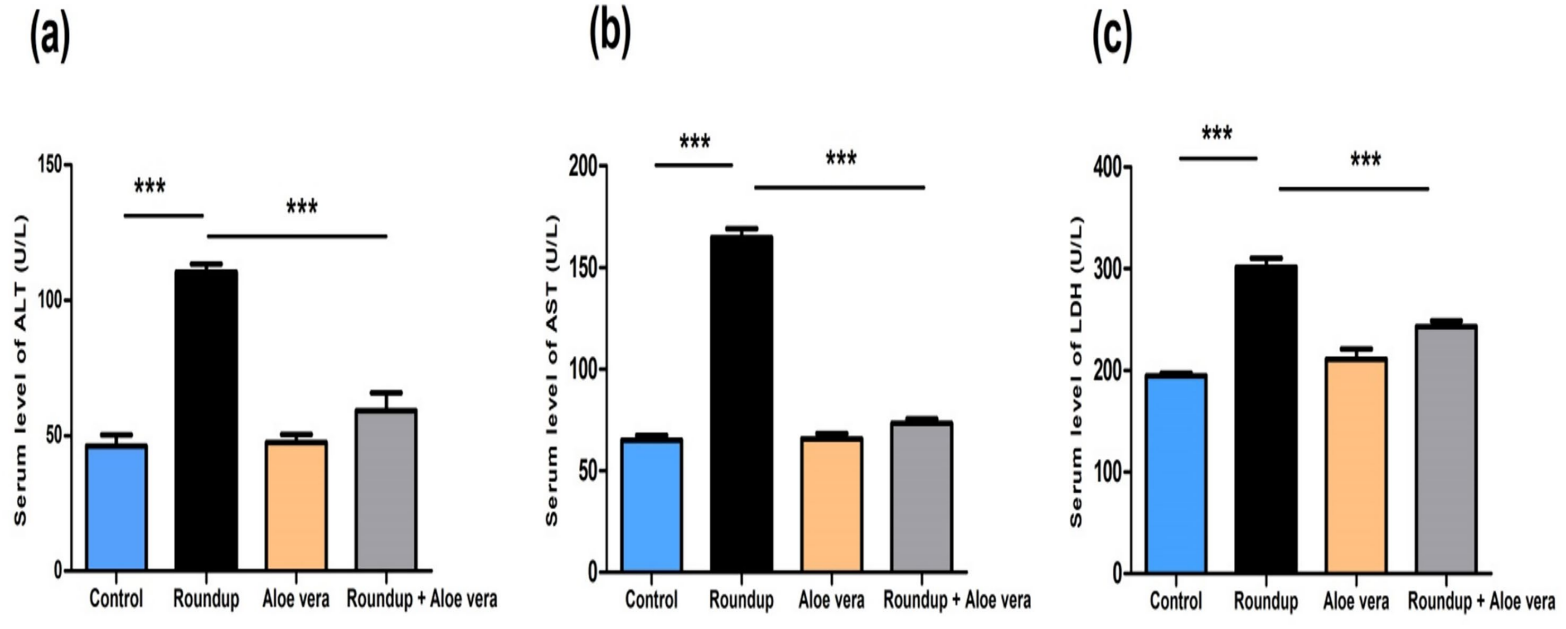
Effect of co-administration of Roundup and *Aloe Vera* on liver function enzymes. Serum levels of **(a)** ALT, **(b)** AST, and **(c)** LDH showing significant down-regulation in the Roundup + *Aloe Vera*-treated rats compared to the Roundup-exposed rats. ***p < 0.001.

Roundup-exposed rats showed obvious hepatic damage and was expressed by significant (*p* < 0.001) up-regulation in serum liver enzymes (ALT, AST, and LDH) levels relative to the control rats. *Aloe Vera* caused a non-significant alteration in liver enzymes levels compared to the control rats. The Roundup + *Aloe Vera*-treated rats had a significant (*p* < 0.001) down-regulation in the ALT, AST, and LDH levels in comparison with the Roundup-exposed rats (Figure 3).

### 3.4 Hepatic DNA damage

**Figure 4:**
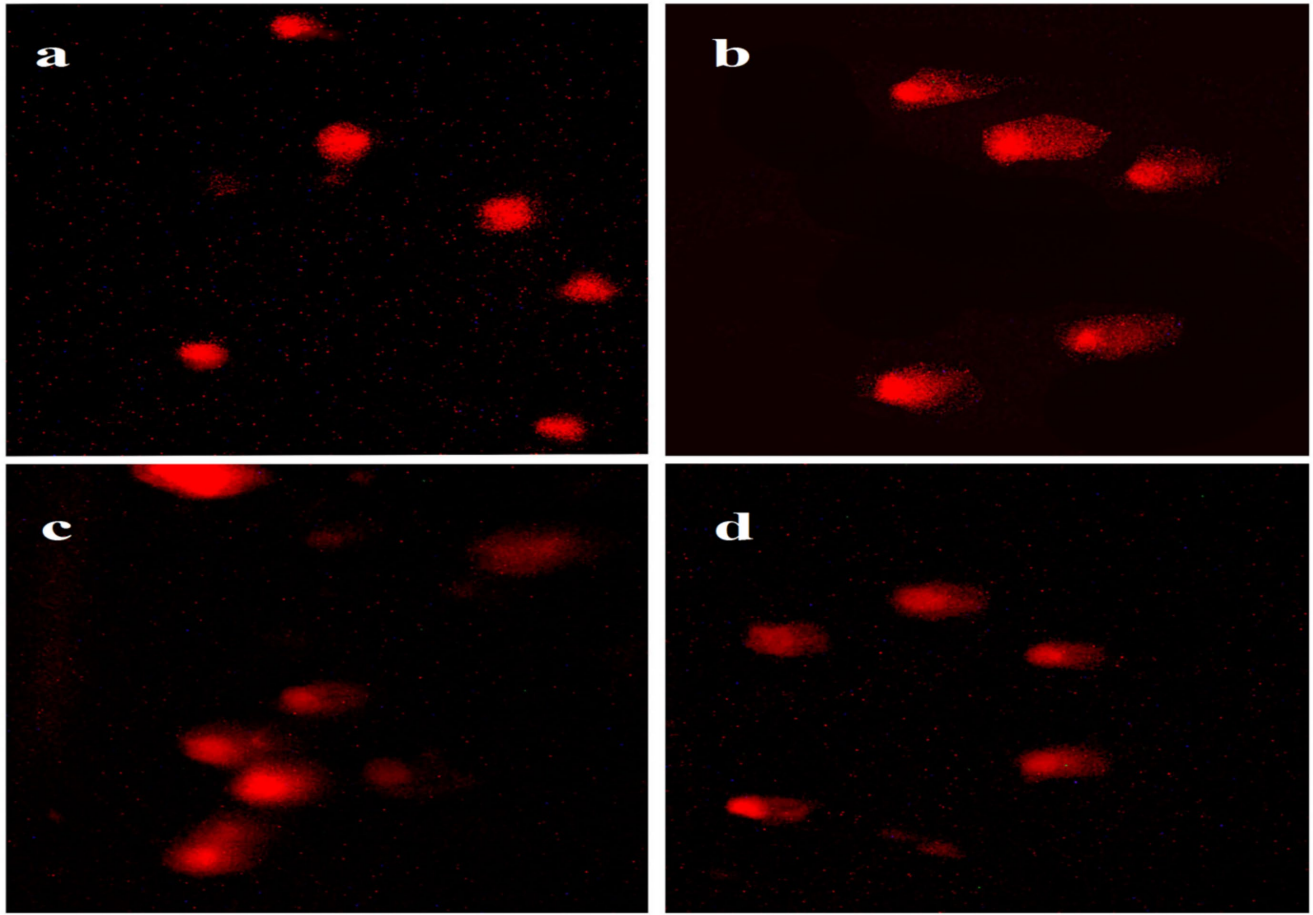
Hepatocyte DNA damage in different groups compared with the control, **(a)** Control, **(b)** Roundup group, **(c)** *Aloe Vera* group and **(d)** Roundup + *Aloe Vera*.

Results showed a non-significant mitigated effect of *Aloe Vera* against hepatic DNA damage of Roundup. Rats which were administered Roundup-based herbicide showed a significant increase in tail moment (23.52), tail length (44.52) and tail DNA % (43.85) of hepatocytes in comparison with the control tail moment (2.07), tail length (8.01) and tail DNA % (8.15), according to Table 1 and Figure 4.

**Table 1:** The role of *Aloe Vera* against the genotoxicity of Roundup herbicide through comet assay parameter in the liver of rats.

Comet assay parameters	Mean ± SE	Control	Round up	*Aloe Vera*	Roundup + *Aloe Vera*
Tail moment	Mean ± SE	2.07 ± 0.81	23.52 ± 3.02a	12.26 ± 2.60ab	15.47 ± 4.63a
Tail length (μm)	Mean ± SE	8.01 ± 5.11	44.52 ± 4.81a	32.23 ± 5.46ab	36.31 ± 4.61a
Tail DNA %	Mean ± SE	8.15 ± 1.90	43.85 ± 3.63a	30.92 ± 2.32ab	35.87 ± 3.44a

*Aloe Vera* mitigated the Roundup-induced genotoxicity. Results are expressed as mean ± SEM (*n* = 6/group). Letters a, b, and c indicate the significant differences between the exposed groups of the experiment, where (a) indicates a significant difference with the control and (b) indicates a significant difference with the Roundup group.

Rats administered with *Aloe Vera* showed a significant decrease in tail moment (12.26), tail length (32.23) and tail DNA % (30.92) in comparison with the Roundup group tail moment (23.52), tail length (44.52) and tail DNA % (43.85); However, they showed a significant increase in comet assay parameters of hepatocytes compared to the control tail moment (2.07), tail length (8.01) and tail DNA % (8.15), according to Table 1 and Figure 4.

Co-administration of *Aloe Vera* with Roundup displayed a significant increase in tail moment (15.47), tail length (36.31) and DNA % (35.87) in comparison with the control tail moment (2.07), tail length (8.01) and DNA % (8.15), according to Table 1 and Figure 4.

### 3.5 Histopathological findings

**Figure 5:**
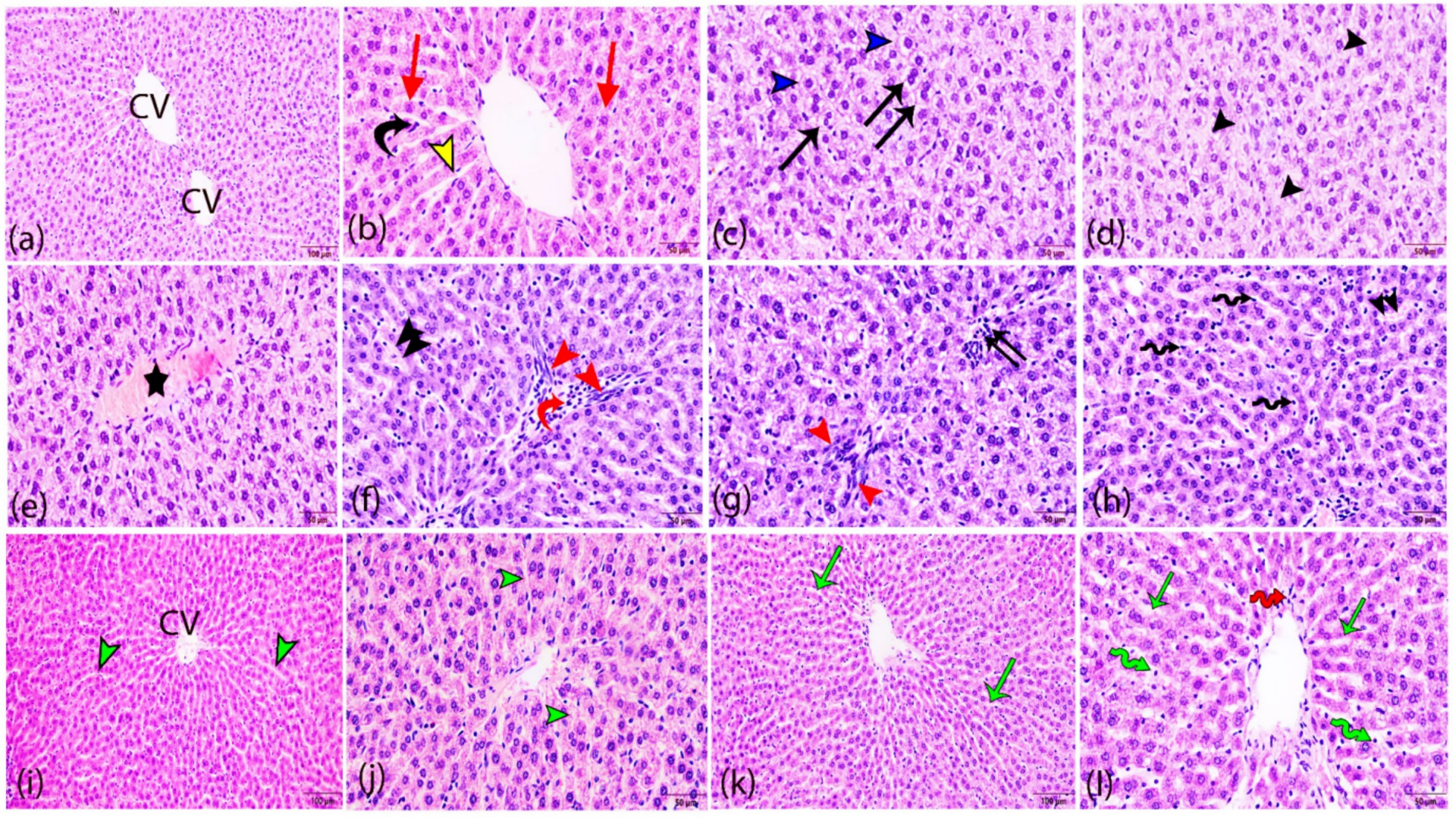
Histopathological analysis of hepatic tissue sections from all studied groups. **(a,b)** Control rats revealing normal hepatocytes (red arrows) radiating from the central vein (CV), blood sinusoids (yellow arrowhead) separating the hepatic plates and containing Kupffer cells (black curved arrow). **(c–h)** Roundup intoxicated group revealing increased binucleated hepatocytes (black arrows), cytoplasmic vacuolation (blue arrowheads), necrosis of sporadic hepatocytes with karyolysis (black arrowheads), congested central vein (star), mononuclear cells infiltration (red curved arrow), oval cell proliferation (red arrowheads), focal neutrophils aggregation (black double arrows), reactive Kupffer cells (zigzag arrows) and congestion and dilation of hepatic sinusoids (double arrowheads). **(I,j)** *Aloe Vera* treated group revealing slight congestion of central vein (cv) and hepatic sinusoids (green arrowheads). **(k,l)** The Roundup + *Aloe Vera*-treated group revealing slight dilation of hepatic sinusoids (green arrows), activation of Kupffer cells (green zigzag arrow) and perivascular infiltration of few inflammatory cells (red zigzag arrow). H&E stain. (**a,i,k**; bar = 100 μm; **b–h**,**j**,**I**; bar = 50 μm).

The score of severity and incidence of histopathological findings is demonstrated in Table 2. Histopathological evaluation of the control rat’s sections of the hepatic tissue revealed normal hepatic morphology with hepatocytes which are arranged in plates radiating outwards from a central vein with sinusoids inbetween. The hepatocytes showed eosinophilic cytoplasm with vesicular central nuclei (Figures 5a,b). In contrast, obvious histopathological changes were evident in the liver sections of Roundup-intoxicated rats. Most hepatocytes were binucleated and displayed cytoplasmic vacuolation. Also, necrosis of sporadic hepatocytes was observed in the same intoxicated group (Figures 5c,d). Vascular changes were also evident, such as congestion of central veins (Figure 5e) and hepatic sinusoids. Mononuclear cells and neutrophils infiltration was seen in the portal area and within hepatic parenchyma. Additionally, Kupffer cell’s reaction and proliferation of oval cells between hepatic cords and in the portal area was noticed (Figures 5f–h).

**Table 2:** Score of severity and incidence of histopathological findings in all experimental groups.

Lesion	^a^Severity score and ^b^incidence of lesions			
	Control	Roundup	*Aloe vera*	Roundup + *Aloe vera*
Vascular congestion & dilatation	^a^0^b^ (0)	3 (6)	1 (2)	1 (5)
Cytoplasmic vacuolation	0 (0)	4 (6)	0 (0)	0 (0)
Necrosis of hepatocytes	0 (0)	3 (6)	0 (0)	0 (0)
Inflammatory cells infiltration	0 (0)	2 (6)	0 (0)	1 (2)
Oval cells proliferation	0 (0)	2 (6)	0 (0)	0 (0)
Kupffer cell’s reaction	0 (0)	2 (6)	0 (0)	1 (1)

a Score of lesion severity (0–4).

b Incidence of lesion (number of rats with lesion per total inspected).

The *Aloe Vera* treated group showed normal histological architecture of hepatic parenchyma with slight congestion of the central vein and hepatic sinusoids in some inspected sections (Figures 5i,j). The Roundup + *Aloe vera* treated group showed noticeable improvement of hepatic parenchymal appearance with slight lesions in the form of dilatation of hepatic sinusoids, reactive Kupffer cells, and perivascular infiltration of few mononuclear cells (Figures 5k,l).

### 3.6 Immunohistochemical detection of apoptotic cells

**Figure 6:**
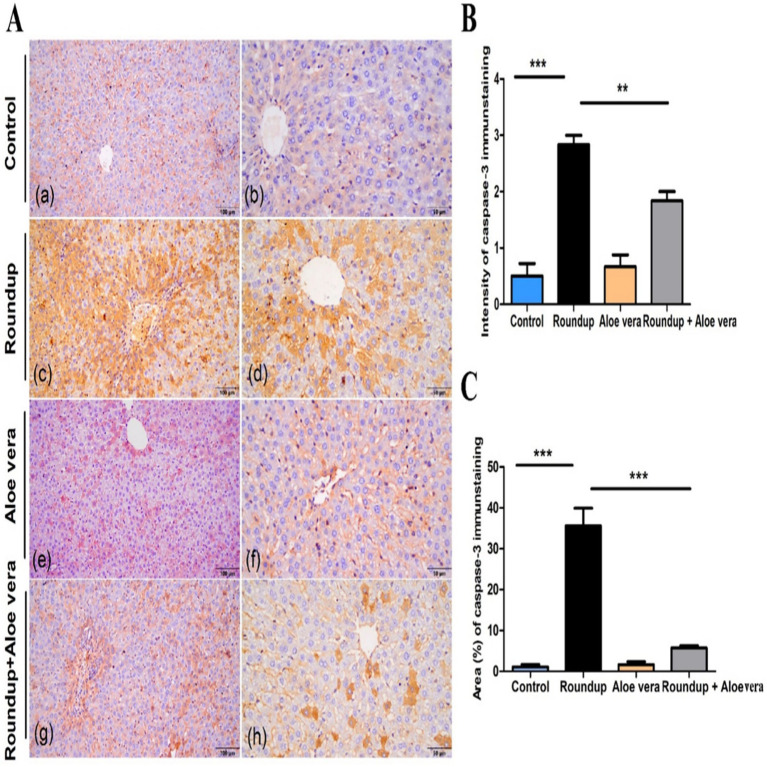
Immunohistochemical analysis of caspase-3 in hepatic tissues. **(A)** Immunoexpression of caspase-3 in Control, Roundup, *Aloe Vera*, and Roundup + *Aloe Vera*. **(B)** Intensity of the immunostaining of caspase-3. **(C)** Area percentage of caspase-3 immunostaining from the total number of pixels. **p < 0.01, ***p < 0.001.

Immunohistochemical analysis of caspase-3 in hepatic tissue sections of various studied groups was displayed in (Figure 6A), where the immunopositively stained hepatocytes revealed a brown-colored cytoplasmic reaction. The hepatic parenchyma of the control had few sporadic hepatocytes with mild immunointensity. In the Roundup group, there were high numbers of immunoreactive hepatocytes with strong intensity widely distributed in the hepatic parenchyma of the stained sections. The *Aloe Vera* group showed few immunostained hepatocytes with mild intensity for caspase-3. The immunoreaction in the Roundup + *Aloe Vera*-treated group involved moderate numbers of hepatocytes in the periportal and centrilobular areas, revealing moderate intensity. The intensity and the area percentage of caspase-3 immunostaining are demonstrated in (Figures 6B,C), respectively.

## 4 Discussion

Roundup is the most commonly and widely used GBH throughout the world (63). In recent years, it has gained more attention, and some countries have started to restrict its usage due to its adverse toxic effects on humans and aquatic organisms (64, 65). It was observed that the Roundup® herbicide alters the liver functions in albino rats through various biochemical, molecular, histopathological and immunohistochemical results. Therefore, the mechanisms and causes of these effects will be addressed and discussed during the current study, in addition to looking at previous studies.

In general, pesticide exposure induces reactive oxygen species (ROS) production, which is responsible for proteins, lipids, and DNA oxidative damage (66). In the current study, a significant elevation in MDA level and a significant exhaustion of GSH levels confirm the potential of Roundup to induce oxidative stress in the liver of rats. Our results are consistent with those in the research of Turkmen et al. (10), who observed an increase in MDA levels in rats which were exposed to sub-chronic oral glyphosate-based herbicide. Hamdaoui et al. (67) showed the ability of Kalach 360 SL to induce liver damage and increase the level of liver lipid peroxidation in rats. Hashim et al. (68) showed that glyphosate led to a significant elevation in MDA levels and decreased the total antioxidant capacity in serum of rats. Moreover, Djaber et al. (36, 37) revealed the adverse effects of Roundup on the antioxidant defense system through the reduction of different antioxidant enzymes and the increase of liver glutathione peroxidase activity in rats.

Oxidative stress is the first suggested mechanism of GBH toxicity (12) due to its toxic effect on mitochondria, which are considered the major cellular sites of ROS production (69). The production of high levels of ROS due to GBH exposure can activate the body enzymes and endogenous antioxidants as defense mechanisms (70), but there is an imbalance between the free radicals’ production and the antioxidant system (10, 68, 71, 72), so the body becomes unable to neutralize the ROS, and oxidative stress occurs. Furthermore, glyphosate-based herbicides act as a glycine analog; during the protein synthesis, they may be mistakenly incorporated into peptides and disrupt the physiological functions which depend on glycine, especially glutathione biosynthesis. Glycine acts as a precursor of glutathione, which plays a vital role in the antioxidant mechanism against free radicals. It is also regulating cytokine production, which in turn can affect the production of free radicals as well as the immune response of the immune system. It is essential for proteins, collagen and elastin synthesis of joints, bones, muscles and cells, increasing their flexibility and endurance (73).

The Roundup-treated group had a significant increase in the tissue levels of TNF-*α* and IL-1β versus the control group. Our findings were in harmony Pandey et al. (35), who found that TNF-α and IL-1β were up regulated in the liver of rats exposed to 100 and 250 mg/kg bw of Roundup. Ngatuni et al. (74) confirmed that GBH increased the TNF-*α* in the serum of mice. Qi et al. (75) showed that glyphosate-based herbicide disrupted the liver functions and structure through the activation of the inflammatory responses through oxidative stress in the liver of mice.

Inflammatory reaction is the second mechanism of GBH’s toxicity, which may be due to its oxidative stress induction and/or its metal chelation ability. Glyphosate strongly chelates calcium, zinc, iron, cobalt, copper, and molybdenum (76, 77), causing various physiological action disturbances. Calcium chelation disrupts the normal physiological cellular processes, including the growth, proliferation, differentiation, and apoptosis (78), where it acts as the most important regulator of apoptosis at all its stages (79). Zinc chelation disrupts different structural and cellular functions (80–82). Zinc deficiency increases the proinflammatory cytokines’ production, such as IL-1β, IL-6, and TNF-α, as well as induces oxidative stress due to its scavenging properties against reactive oxygen species (ROS) (83). Chelation of iron causes inactivation of cytochrome P450 enzymes (84), which play a vital role in neurosteroid production to prevent cellular stress (85). Glyphosate also acts as a chelating agent to copper (86), which is considered a vital element and antioxidant in the body.

The leakage of hepatic enzymes to circulation is considered the common indirect biochemical index of hepatocellular injury (87). Statistically, in the current study ALT, AST, and LDH enzymes showed a significant increase in the serum of rats after 6 weeks of exposure to Roundup compared to the control. This observation was in line with Saleh et al. (34), who observed an increase in ALT and AST levels in the serum of rats exposed to Roundup® for 15 days. Dar et al. (88) reported an elevation in the activities of AST, ALT, and LDH in Wistar rats’ serum. Tizhe et al. (89) showed the increase in serum enzymes (AST, ALT, and ALP), which related to liver damage in rats following oral exposure to glyphosate-based herbicide. Hashim et al. (68) showed a significant increase in ALT and AST enzyme activities in the serum of rats which were exposed to Roundup herbicide. Djaber et al. (36, 37) revealed the significant increase in levels of AST, ALT, LDH, ALP and GGT liver enzymes in Roundup-exposed rats. Release of the hepatic enzymes into the circulation after glyphosate-based herbicide exposure may be related to the cell membrane damage due to the high level of free radical production, exhaustion of the antioxidant systems (90, 91) and the hepatic inflammation (35, 92).

The comet assay acts as a biomarker used for genotoxicity detection, assessment and measurement of DNA damage (93, 94). During the current study, it was worth noticing that glyphosate-based herbicides caused DNA damage in liver cells, where the Roundup-intoxicated rats had a significant increase in tail moment, length and DNA % of hepatocytes compared to the control. Our findings were in harmony with Milić et al. (95), who observed hepatocytes’ and leucocytes’ DNA damage in Wistar rats exposed to glyphosate for 28 days. DNA damage occurrence may be secondary to liver oxidative stress and/or inflammation induced by glyphosate-based herbicides.

Various histopathological lesions were observed in the liver of Roundup-intoxicated rats, where most of the hepatocytes showed necrosis and binucleated with cytoplasmic vacuolation, as well as vascular changes such as congestion of the central vein and hepatic sinusoid accompanied by periportal mononuclear cell infiltration. There were proliferation of oval cells between hepatic cords and in the portal area, and small leucocytic aggregation among hepatocytes, as well as dilation of hepatic sinusoids with activation of Kuffer cells. Our findings were in harmony with the studies which were conducted by Dar et al. (88) and observed moderate hepatocytic degeneration and necrosis in the liver of rats which were exposed to 500 mg/kg b.wt. of Roundup herbicide. Saleh et al. (34) showed the histopathological lesions of different doses of Roundup® in albino rats. Pandey et al. (35) showed the formation of fibroid tissue and vacuoles, as well as depletion of glycogen in the liver of rats treated with high doses of Roundup. Tizhe et al. (89) observed the mononuclear cell infiltration, fatty degeneration and coagulative necrosis of hepatocytes in the liver of rats exposed to Bushfire VR (one of GBHs). Hashim et al. (68) observed several histopathological alterations in liver sections of glyphosate-exposed rats compared to the control. Djaber et al. (36, 37) revealed severe structural damage of the liver of Roundup-treated rats.

Immunohistochemical analysis of caspase-3 in hepatic tissue sections of the Roundup group showed that high numbers of immunoreactive hepatocytes with strong intensity were widely distributed in the hepatic parenchyma of the stained sections compared to the control. This observation was in agreement with Hashim et al. (68), showing that glyphosate caused overexpression of apoptotic markers in adult male albino rats. Glyphosate-based herbicides produce high levels of free radicals, which react rapidly with the cellular component, producing biochemical, physiological and histological lesions which lead to apoptosis.

Co-administration of *Aloe Vera* with Roundup ameliorates the biochemical, histopathological, and immunohistochemical alterations following oral exposure to Roundup herbicide for 6 weeks in albino rats. These findings suggested that *Aloe Vera*’s ameliorative effects were attributed to various pharmacological actions as an antioxidant and scavenger of free radicals (96) and anti-inflammatory (51, 97) properties thanks to its content of a myriad of bioactive compounds. The current study is considered the first approach to evaluate the effectiveness of *Aloe Vera*-based medicines against GBH toxicity. Ameliorative effect of *Aloe Vera* against pesticide toxicity was reported previously by Gupta et al. (98), who observed the ameliorative role of *Aloe Vera* against Cartap-induced neurotoxicity in Wistar rats. Gupta et al. (99) showed the antioxidant effect of *Aloe Vera* against malathion neurotoxicity in rats.

There are various plant antioxidants, which have been studied previously against glyphosate-based herbicides toxicity in rats, and can be compared to the *Aloe Vera* that used during the current study where, *Morus alba* Leaf Extract alleviated Roundup- induced oxidative stress and inflammation in liver of rats (100). *Linum usitatissimum* oil had ameliorative effects against hepatic and renal toxicity of Roundup based herbicide (36). Finally, Resveratrol had a protective role against oxidative stress, and histopathological lesions induced by Knockdown 48 SL as a one of glyphosate-based herbicide in rats (10).

On the other hand, the current study results showed that *Aloe Vera* caused a non-significant ameliorative effect on hepatocyte DNA damage induced by Roundup, which may be due to the imbalance between the free radicals’ production with GBHs and the antioxidant mechanism of *Aloe Vera*, or it has a genotoxic effect. Also, the authors suggested that the DNA damage which was observed in rats exposed to *Aloe Vera* only is not related to oxidative stress; no oxidative stress occurred in the *Aloe Vera* group. These results indicated that the genotoxicity of *Aloe Vera* must be studied in depth in further studies to determine the dose which is required to give the best improvement. Although *Aloe Vera* has valuable properties and variable oral or topical medical applications (101), it is no longer safe and effective (102). The International Agency for Research on Cancer classified it as a possible human carcinogen (Group 2B) (103, 104).

## 5 Conclusion

In conclusion, the current results indicated that exposure of rats to Roundup herbicide resulted in determined biochemical (MDA, GSH, ALT, AST, LDH, IL-1β and TNF-*α*), molecular (DNA damage), histopathological and immunohistochemical (caspase-3) alterations in the liver. *Aloe Vera* can ameliorate most of the adverse effects of GBHs thanks to its antioxidant and anti-inflammatory properties. The persistence of DNA damage may be related to the genotoxic effect of *Aloe Vera* itself. The current study is limited to the ameliorative role of single dose of *Aloe Vera* against Round up-based herbicide hepatotoxicity and considered the cornerstone of further future studies, which needed to (1) conduct chronic studies with variable dosages to assess the *Aloe Vera*-potential adverse effects or safety over time and confirm its carcinogenic and genotoxic actions. (2) Evaluate the toxic effect of Round up-based herbicide and the role of *Aloe Vera* in different body systems, especially renal, nervous and genital systems. (3) Finally, to confirm the results in another species and sex, where the current study was specific to female Sprague–Dawley rats.

## Data Availability

The original contributions presented in the study are included in the article/supplementary material, further inquiries can be directed to the corresponding authors.
